# Supplementary material for: Mapping the evolution of fertility support policies in China: A content and instrumental analysis
Source: PLoS One. 2025 Oct 9;20(10):e0332137. doi: 10.1371/journal.pone.0332137 (PMC12510515; doi:10.1371/journal.pone.0332137)
Supplement: S1 Appendix — (ZIP) [file pone.0332137.s001.zip › S1 Appendix. 226 original policy documents/195-国家发展改革委、国务院妇儿工委办公室、住房城乡建设部等关于推进儿童友好城市建设的指导意见(FBM-CLI-4-5077634).doc]

国家发展改革委、国务院妇儿工委办公室、住房城乡建设部等关于推进儿童友好城市建设的指导意见

**发布部门：** [国家发展和改革委员会(含原国家发展计划委员会、原国家计划委员会)](https://www.pkulaw.com/law/chl?Aggs.IssueDepartment=60301) [国务院妇女儿童工作委员会](https://www.pkulaw.com/law/chl?Aggs.IssueDepartment=7060250) [住房和城乡建设部](https://www.pkulaw.com/law/chl?Aggs.IssueDepartment=60238) [中共中央宣传部](https://www.pkulaw.com/law/chl?Aggs.IssueDepartment=a04) [中央网络安全和信息化委员会办公室](https://www.pkulaw.com/law/chl?Aggs.IssueDepartment=a23) [教育部](https://www.pkulaw.com/law/chl?Aggs.IssueDepartment=60203) [公安部](https://www.pkulaw.com/law/chl?Aggs.IssueDepartment=60205) [民政部](https://www.pkulaw.com/law/chl?Aggs.IssueDepartment=60208) [财政部](https://www.pkulaw.com/law/chl?Aggs.IssueDepartment=60210) [自然资源部](https://www.pkulaw.com/law/chl?Aggs.IssueDepartment=60243) [生态环境部](https://www.pkulaw.com/law/chl?Aggs.IssueDepartment=60242) [交通运输部](https://www.pkulaw.com/law/chl?Aggs.IssueDepartment=60235) [文化和旅游部](https://www.pkulaw.com/law/chl?Aggs.IssueDepartment=60241) [国家卫生健康委员会](https://www.pkulaw.com/law/chl?Aggs.IssueDepartment=60321) [应急管理部](https://www.pkulaw.com/law/chl?Aggs.IssueDepartment=60244) [国家市场监督管理总局](https://www.pkulaw.com/law/chl?Aggs.IssueDepartment=60426) [国家广播电视总局](https://www.pkulaw.com/law/chl?Aggs.IssueDepartment=60427) [国家体育总局](https://www.pkulaw.com/law/chl?Aggs.IssueDepartment=60407) [国家医疗保障局](https://www.pkulaw.com/law/chl?Aggs.IssueDepartment=60431) [国家林业和草原局](https://www.pkulaw.com/law/chl?Aggs.IssueDepartment=60428) [共青团中央](https://www.pkulaw.com/law/chl?Aggs.IssueDepartment=70402) [全国妇女联合会](https://www.pkulaw.com/law/chl?Aggs.IssueDepartment=70403) [中国残疾人联合会](https://www.pkulaw.com/law/chl?Aggs.IssueDepartment=7060804) [机构沿革](javascript:void(0))

**发文字号：**发改社会〔2021〕1380号

**发布日期：**2021.09.30

**实施日期：**2021.09.30

**时效性：** [现行有效](https://www.pkulaw.com/law/chl?Aggs.TimelinessDic=01)

**效力级别：** [部门规范性文件](https://www.pkulaw.com/law/chl?Aggs.EffectivenessDic=XE0302)

**法规类别：** [城市规划与开发建设](https://www.pkulaw.com/law/chl?Aggs.Category=04903) [老少妇幼残保护](https://www.pkulaw.com/law/chl?Aggs.Category=01604)

国家发展改革委、国务院妇儿工委办公室、住房城乡建设部、中央宣传部、中央网信办、教育部、公安部、民政部、财政部、自然资源部、生态环境部、交通运输部、文化和旅游部、国家卫生健康委、应急部、市场监管总局、广电总局、体育总局、国家医保局、国家林草局、共青团中央、全国妇联、中国残联关于推进儿童友好城市建设的指导意见
（发改社会〔2021〕1380号）

各省、自治区、直辖市人民政府，新疆生产建设兵团：
　　儿童友好是指为儿童成长发展提供适宜的条件、环境和服务，切实保障儿童的生存权、发展权、受保护权和参与权。建设儿童友好城市，寄托着人民对美好生活的向往，事关广大儿童成长发展和美好未来。为落实党中央、国务院决策部署，推进儿童友好城市建设，让儿童成长得更好，经国务院同意，提出以下意见。
　　一、总体要求
　　**（一）**指导思想
　　坚持以习近平新时代中国特色社会主义思想为指导，全面贯彻党的十九大和十九届二中、三中、四中、五中全会精神，坚持以人民为中心的发展思想，坚持以立德树人为根本，坚持儿童优先发展，从儿童视角出发，以儿童需求为导向，以儿童更好成长为目标，完善儿童政策体系，优化儿童公共服务，加强儿童权利保障，拓展儿童成长空间，改善儿童发展环境，全面保障儿童生存、发展、受保护和参与的权利，让儿童友好成为全社会的共同理念、行动、责任和事业，让广大儿童成长为德智体美劳全面发展的社会主义建设者和接班人，不断为实现中华民族伟大复兴的中国梦贡献力量。

　　**（二）**基本原则
　　--儿童优先，普惠共享。坚持公共事业优先规划、公共资源优先配置、公共服务优先保障，推动儿童优先原则融入社会政策。坚持公益普惠导向，扩大面向儿童的公共服务供给，让广大适龄儿童享有公平、便利、安全的服务。
　　--中国特色，开放包容。立足国情和发展实际，促进儿童参与，探索中国特色儿童友好城市建设路径模式。结合推进“一带一路”建设，坚持世界眼光，借鉴有益经验，强化交流互鉴，以儿童友好促进民心相通。
　　--因地制宜，探索创新。适应城市经济社会发展水平，结合资源禀赋特点，因城施策推进儿童友好城市建设。鼓励有条件的城市改革创新，先行先试，探索建设模式经验，积极发挥示范引领作用。
　　--多元参与，凝聚合力。坚持系统观念，强化儿童工作“一盘棋”理念，发挥党委领导、政府主导作用，健全完善多领域、多部门工作协作机制，积极引入社会力量，充分激发市场活力，形成全社会共同推进儿童友好城市建设的合力。

　　**（三）**建设目标
　　到2025年，通过在全国范围内开展100个儿童友好城市建设试点，推动儿童友好理念深入人心，儿童友好要求在社会政策、公共服务、权利保障、成长空间、发展环境等方面充分体现。展望到2035年，预计全国百万以上人口城市开展儿童友好城市建设的超过50%，100个左右城市被命名为国家儿童友好城市，儿童友好成为城市高质量发展的重要标识，儿童友好理念成为全社会共识和全民自觉，广大儿童享有更加美好的生活。
　　二、推进社会政策友好，推动全社会践行儿童友好理念

　　**（四）**推动儿童优先发展。制定城市经济社会发展规划优先考虑儿童需求，推进公共资源配置优先满足儿童需要。健全推进儿童优先发展工作协调机制，重点在政策协调、资金投入、项目实施等方面形成合力。在城市发展重大规划、政策、项目决策中引入儿童影响评价。探索应用数字化手段创新儿童工作方式方法。

　　**（五）**城市规划建设体现儿童视角。引入“1米高度看城市”儿童视角，推进儿童友好理念融入城市规划建设。制定城市各类儿童友好空间与设施规划建设标准，完善城市功能布局，优化公共空间设计，推进城市建设适应儿童身心发展，满足儿童服务和活动需求。

　　**（六）**推动儿童全方位参与融入城市社会生活。建立健全儿童参与公共活动和公共事务机制，畅通儿童参与渠道，涉及儿童的重大事项事先听取儿童及监护人意见。在制定社会政策、发展公共服务中尊重儿童的独立人格，全面保障儿童在社会生活、社区发展、家庭事务中的知情权、表达权和参与权。

　　**（七）**发动全社会力量共同致力儿童发展。坚持以普惠为导向，鼓励政府、企事业单位、家庭和个人参与，整合全社会资源增进儿童福祉。积极培育为儿童服务的社会组织、专业社会工作者、少先队校外辅导员和志愿者队伍，发展儿童公益慈善事业。
　　三、推进公共服务友好，充分满足儿童成长发展需要

　　**（八）**支持发展普惠托育服务。鼓励支持企事业单位和社会组织、社区等提供普惠托育和婴幼儿照护服务。探索实施父母育儿假制度，加强家庭科学育儿指导服务。

　　**（九）**促进基础教育均衡发展。完善普惠性学前教育保障机制，进一步提高普及普惠水平，严格落实城镇小区配套幼儿园政策，补齐资源短板，提高保教质量。完善义务教育免试就近入学制度，精准做好控辍保学，保障流动儿童平等接受教育权利。加强特殊教育资源建设，“一人一案”做好适龄残疾儿童入学安置。落实政府举办义务教育的主体责任，强化学校教育主阵地作用，提高学校课堂教学质量，全面压减作业总量和时长，提升学校课后服务水平，全面规范校外培训行为，进一步减轻义务教育阶段学生作业负担和校外培训负担。开展儿童友好学校建设。

　　**（十）**加强儿童健康保障。关注生命早期1000天健康保障，加强婚前、孕前、孕产期保健和儿童早期发展服务。推进实施出生缺陷综合防治、母乳喂养促进行动，提高优生优育服务水平，建设母婴友好医院。建设儿童保健服务网络，做好儿童健康管理，规范预防接种和防治龋齿，降低近视及肥胖发生率。关注儿童心理健康，开展儿童生命教育、性教育，培养珍爱生命意识，提升自我情绪调适能力。

　　**（十一）**服务儿童看病就医和医疗保障。以儿童医院、妇幼保健机构、综合医院儿科、乡镇卫生院、社区卫生服务中心为重点，以儿科和儿童保健科为支撑，加强儿童医疗服务网络建设，提供优质诊疗服务。加强新生儿科等儿科医师培训，完善相关人才发展激励机制。强化基本医保、大病保险与医疗救助三重制度综合保障功能，做好儿童基本医疗保障工作。开展儿童友好医院建设。

　　**（十二）**丰富儿童文体服务供给。合理规划文体设施布局和功能，推进图书馆、文化馆、美术馆等向儿童免费开放，推动有条件的公共体育设施向儿童低收费或免费开放，组织面向儿童的阅读推广、文艺演出、展览游览等活动。制修订面向儿童的体育设施器材等标准，加强儿童体质监测，丰富儿童体育赛事活动，加强从业人员队伍建设。
　　四、推进权利保障友好，完善公益普惠儿童福利体系

　　**（十三）**关爱孤儿和事实无人抚养儿童。深化孤儿助医助学项目，探索推进事实无人抚养儿童助医助学，优化完善社会散居孤儿、事实无人抚养儿童家庭走访、监护评估、家庭培训和监护保护制度。完善儿童收养登记管理，推进实施收养评估制度。推进儿童福利机构优化提质，支持建造家庭式居所，推广家庭式养育模式。

　　**（十四）**推进残疾儿童康复服务。协同推进儿童残疾预防、早期筛查、诊疗康复，鼓励有条件的地方扩大残疾儿童康复救助项目及年龄范围，提高救助标准，鼓励公办机构开展康复业务，支持社会力量举办康复机构，加强康复救助定点服务机构管理。

　　**（十五）**加强困境儿童分类保障。建立健全困境儿童信息台账，落实定期上门查访，加大对困难家庭的重病、残疾儿童基本生活保障和专项救助力度。落实孤儿、留守儿童、困境儿童基本医疗保障政策，实施分类救助。加强流浪未成年人救助保护。
　　五、推进成长空间友好，提升城市空间品质和服务效能

　　**（十六）**推进城市公共空间适儿化改造。加强城市街区、社区、道路以及学校、医院、公园、公共图书馆、体育场所、绿地、公共交通等各类服务设施和场地适儿化改造。建设适合儿童的服务设施和标识标牌系统，推动公共场所建设母婴室、儿童厕位及洗手池、儿童休息活动区等。加强儿童友好街区建设。

　　**（十七）**改善儿童安全出行体验。完善慢行交通体系，加强人行道、自行车道规划建设，优化校园周边步行线路规划和人行设施，保障儿童出行安全。加快完善城市公共交通场站、过街无障碍设施。加强交通安全教育，增强儿童安全出行能力。

　　**（十八）**拓展儿童人文参与空间。拓展儿童阅读空间，在公共图书馆设置儿童阅览区，鼓励设置少儿图书馆，提供适宜残疾儿童的阅读资源，开展儿童友好图书馆建设。扩充儿童美育资源，鼓励学校与美术馆、博物馆、音乐厅等共建校外教育基地。增加儿童校外活动空间，加强儿童劳动教育、课外实践、科技体验、素质拓展等校外活动场所设施建设。

　　**（十九）**开展儿童友好社区建设。建设社区儿童之家等公共空间，为儿童提供文体活动和阅读娱乐场所。增加社区儿童“微空间”，鼓励社区打造儿童“游戏角落”，提供适龄儿童步行路径和探索空间，合理增设室内外安全游戏活动设施。

　　**（二十）**开展儿童友好自然生态建设。建设健康生态环境，推动开展城市儿童活动空间生态环境风险识别与评估评价。推动建设具备科普、体验等多功能的自然教育基地。开展儿童友好公园建设，推进城市和郊野公园设置游戏区域和游憩设施，合理改造利用绿地，增加儿童户外活动空间。

　　**（二十一）**提升灾害事故防范应对能力。推动落实儿童密集场所安全主体责任和行业监管责任，有效防范应对各类灾害事故风险。强化防灾减灾安全教育，增强儿童防灾减灾意识和自救互救能力。储备面向儿童需求的重要应急物资。
　　六、推进发展环境友好，优化儿童健康成长社会环境

　　**（二十二）**推进家庭家教家风建设。深入实施家家幸福安康工程，建设文明家庭、实施科学家教、传承优良家风。构建学校家庭社会协同育人体系，加强家庭教育指导服务，增强家庭监护责任意识和能力，建立良好亲子关系，培养儿童良好思想品行和生活习惯。

　　**（二十三）**培养健康向上的精神文化。鼓励创作符合儿童特点的优秀文化产品，加强社会主义核心价值观教育。组织开展优秀传统文化进校园、进课堂活动，深入开展共青团、少先队实践活动。普及发展青少年健身运动，让更多儿童经常性参与体育锻炼。

　　**（二十四）**持续净化网络环境。加强网络环境保护，聚焦网络直播、网络游戏等儿童上网重点环节和应用，及时发现处置危害儿童身心健康的不良信息，严厉查处违法违规行为。加大儿童用户量集中的网络平台日常监管，规范涉儿童相关网站管理，压实互联网企业维护网络环境责任。

　　**（二十五）**筑牢安全发展屏障。开展中小学生安全教育日活动，深化少年警校建设，推进“护校安园”专项行动，加强校园、校舍和校车安全管理。落实食品安全校（园）长负责制，保障在校学生安全营养用餐。强化儿童用品安全监管，加强监督检查，及时向社会公示。

　　**（二十六）**防止儿童意外和人身伤害。健全儿童交通、溺水、跌落、烧烫伤、中毒等重点易发意外事故预防和处置机制。做好学生欺凌防治工作，有效防范性侵、家暴事件，严格落实侵害未成年人案件强制报告制度，保障儿童合法权益。及时受理、依法查处儿童失踪案事件，严厉打击拐卖儿童等犯罪行为。

　　**（二十七）**积极预防未成年人犯罪。推进实施未成年人违法犯罪分级干预机制，及时发现、制止未成年人不良行为和严重不良行为。对涉罪未成年人坚持依法惩戒与精准帮教相结合，增强教育矫治效果，预防重新犯罪。
　　七、组织实施

　　**（二十八）**加强组织领导。国家发展改革委、国务院妇儿工委办公室、住房城乡建设部等统筹协调儿童友好城市建设工作。建立健全儿童友好城市建设、认定、评估、监测等机制，研究儿童友好城市认定办法。行业主管部门按职能开展相关领域儿童友好行动，制定标准体系或建设指南。省级政府有关部门制定本地区儿童友好城市建设实施方案。城市政府履行建设主体责任，整体制定落实建设方案。

　　**（二十九）**完善政策支持。中央财政统筹利用现有资金渠道，发挥中央预算内投资的引导和撬动作用，对儿童友好城市建设予以积极支持。对价格普惠且具有一定收益的儿童服务设施项目，符合条件的可纳入地方政府专项债券支持范围。地方要统筹中央相关转移支付资金和自有财力，强化政策支持。强化公益普惠类儿童服务项目规划用地保障。鼓励地方政府以购买服务、租金减免等方式发展普惠性儿童服务。

　　**（三十）**分批推进建设。制定实施国家儿童友好城市建设行动方案，支持有建设意愿、基础较好的城市先行探索，分批分期滚动推进实施。对于不具备整体建设条件的城市，鼓励从儿童友好社区建设起步，以点带面夯实基础。对建设成效突出的城市命名为国家儿童友好城市。支持有条件的城市发起“一带一路”儿童友好城市联盟。

　　**（三十一）**加强监测评估。组织制定儿童友好城市建设评估指标体系，适时调整完善政策措施。开展儿童友好建设的城市及时收集、整理、分析儿童数据信息，对本地方儿童发展整体情况实施动态监测，探索制定符合地方特点的儿童友好城市建设标准和分领域建设指南，开展建设情况评估。

　　**（三十二）**强化宣传交流。开展儿童友好城市公益宣传，提高公众知晓度和参与度，把儿童友好的理念向全社会推广。总结国内城市建设经验，推广具有重大示范效应的建设模式。借鉴国际有益做法，加强对外交流合作，全面提升中国儿童友好城市国际影响力。

国家发展改革委
国务院妇儿工委办公室
住房城乡建设部
中央宣传部
中央网信办
教 育 部
公 安 部
民 政 部
财 政 部
自然资源部
生态环境部
交通运输部
文化和旅游部
国家卫生健康委
应 急 部
市场监管总局
广电总局
体育总局
国家医保局
国家林草局
共青团中央
全国妇联
中国残联
2021年9月30日

© 北大法宝：（[www.pkulaw.com](https://www.pkulaw.com/)) 专业提供法律信息、法学知识和法律软件领域各类解决方案。北大法宝为您提供丰富的参考资料，正式引用法规条文时请与标准文本核对。
欢迎查看所有[产品和服务](https://www.pkulaw.com/helps/2.html)。法宝快讯：[如何快速找到您需要的检索结果？](https://www.pkulaw.com/helps/70.html)[法宝](https://www.pkulaw.com/helps/69.html)V6有何新特色？

扫描二维码阅读原文

**原文链接：**<https://www.pkulaw.com/chl/a8b7fc5e5d6b38dbbdfb.html>
